# First report of scorpionism caused by *Tityus serrulatus*, described by Lutz and Mello, 1922 (Scorpiones, Buthidae), a species non-native to the state of Pará, Brazilian Amazon

**DOI:** 10.1590/0037-8682-0285-2019

**Published:** 2020-03-16

**Authors:** Gabriela Góes Costa, Luana de Fátima Monteiro Serejo, Johne de Souza Coelho, Denise Maria Cândido, Maria Apolônia da Costa Gadelha, Pedro Pereira de Oliveira Pardal

**Affiliations:** 1 Universidade Federal do Pará, Hospital Universitário João de Barros Barreto, Centro de Ciências da Saúde, Belém, PA, Brasil.; 2 Universidade Federal do Pará, Núcleo de Medicina Tropical, Laboratório de Entomologia médica e Artrópodes Peçonhentos, Belém, PA, Brasil.; 3 Instituto Butantan, Laboratório de Artrópodes, São Paulo, SP, Brasil.

**Keywords:** Scorpionism, Scorpion sting, Brazil

## Abstract

This reports a case of scorpionism caused by *Tityus serrulatus*. A male adult was stung while unloading bananas at the supply center in Belém, Pará, Brazil. The bananas originated in another state (Bahia) and were brought to Belém by truck. The patient presented with pain, edema, and erythema at the sting site, and was classified as low-risk. The specimen was identified as *T. serrulatus* and symptomatic treatment and clinical observation were advised. The patient was discharged later without further complications. This is the first known envenomation caused by *T. serrulatus,* a non-native species to Pará, in the Brazilian Amazon.

## INTRODUCTION

There are 2433 known scorpion species worldwide. The occurrence of envenomation is uncommon from venomous species, with over 1 million envenomations per year, and the World Health Organization have classified scorpion envenomations as a neglected health problem^1^. 

There are approximately 160 recorded species in the Brazilian territory, where *Tityus serrulatus, T. stigmurus, T. bahiensis,* and *T. obscuru*s are associated with higher clinical importance^2^ ^,^ ^3^. *T. serrulatus* is associated with envenomations in the South, Southeast, Midwest, and Northeast regions. This species is absent in the northern region^2^, where envenomation by *T. obscurus, T. metuendes*, and *T. silvestris* prevails^4^ ^,^ ^5^. 

*T. serrulatus* is ranked among the 30 most dangerous scorpion species in the world and is of utmost clinical importance in South America**.** This species is 55-65 cm in length, is dark or light yellow in color, and is known to have three and five spinoid granules on its III and IV metasomas. Its telson has an elliptic vesicle where the aculeus and a prominent subaculear tubercle can be found^6^.

This article describes the first case of envenomation by *T. serrulatus,* a species non-native to the northern region of the Brazilian Amazon and more specifically in the city of Belém, in the Pará State.

## CASE REPORT

**FIGURE 1:**
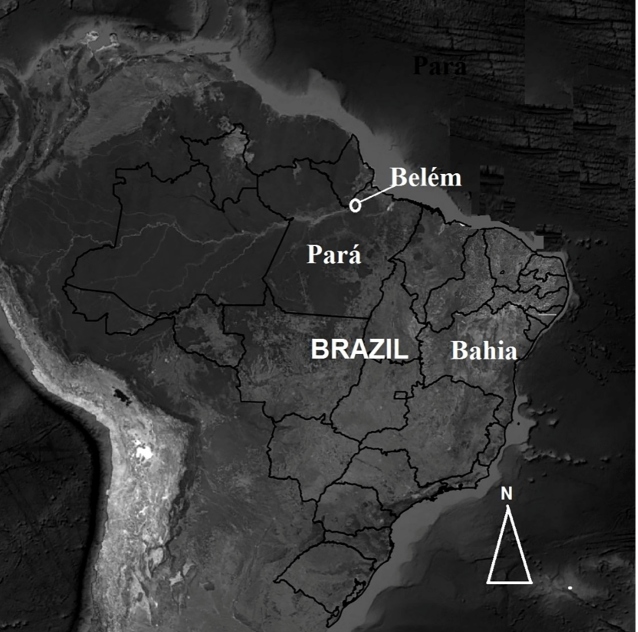
Map of Brazil, highlighting the Pará State and Belém Municipality, the site of the *Tityus serrulatus* stinging incident.

**FIGURE 2:**
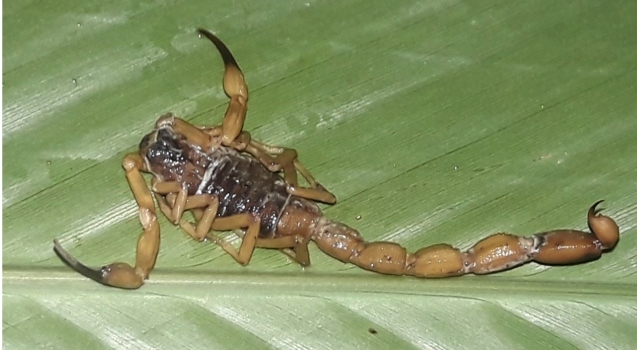
*Tityus serrulatus* scorpion (Lutz & Mello, 1922), responsible for the accident.

A 27-year-old male sales person was stung by a scorpion on the left gluteal region at 2 a.m. on 5t^h^ February 2019. This occurred while the patient was unloading bunches of bananas transported from Bahia by truck at the Supply Centre of Belém, Pará - CEASA/PA (coordinates: 01º 27' 21" S, 48º 30' 16" W) (Figure 1). The patient presented with moderate pain, erythema, and light edema at the sting site. He was referred to clinical care at HPSM Mário Pinotti three hours after the envenomation, bringing the specimen with him for identification. Two ampoules of anti-arachnid serum were administered, and the Belém Toxicological Information Centre was contacted for guidance and species identification. The incident was classified as low-risk; the patient was managed with symptomatic treatment, life support, and active observation for 6 hours. He was discharged after clinical improvement and the scorpion was identified as *T. serrulatus* (Figure 2). 

The ethics committee at the University Hospital João de Barros Barreto, Belém, PA approved this case study under certificate number 09257319.5.0000.0017. The scorpion was given to the care of the Laboratory of Entomology and Venomous Animals, which is a part of the Nucleus of Tropical Medicine at the Federal University of Pará (UFPA).

## DISCUSSION

The *Tityus* genus is associated with most cases of scorpionism in Brazil. Cases are primarily associated with the species *T. serrulatus* and *T. bahiensis* in the East, South, and Midwest regions^7^. *T. stigmurus* and *T. obscurus* are the major causes of stinging incidents in the Northeast and North regions, respectively^4^ ^,^ ^8^. 

Four scorpion families, 12 genera, and 44 species are commonly found in the North region, but *T. serrulatus* is not among them^6^. *T. serrulatus* has parthenogenetic reproduction^2^, which is the ability to reproduce without fertilization and without a co-parent. This implies that a single specimen transported to a new location could reproduce and develop a colony. Road networks, which are the main logistics route in Brazil, are presumably enabling the spread of this species. Lourenço and Eicksteadt^9^ described the species in the state of Rondônia but did not associate it with any clinical cases. This article describes a scorpion-related envenomation caused by a specimen that was carried in fruit cargo. Torres et al.^10^ reported a similar case where a fruit distributor was stung by *T. serrulatus* while handling green peppers that originated in the state of São Paulo.

Pain after a scorpion sting is quite common, and is a hallmark of almost all cases^4^ ^,^ ^7^ ^,^ ^8^. Findings of localized pain, edema, and erythema at the sting site classify the envenomation as low-risk^11^. Common anatomical sites associated with scorpionism are the hands and feet^3^. *T. serrulatus* is the species most associated with high-risk envenomations and lethal cases in Brazil^7^. According to Silva et al.^12^, the low-risk envenomation described here may be due to a regional variation of this species found in the state of Bahia, which is known to have less toxin**s** than other *T. serrulatus* species. 

Antitoxin treatment is based on the risk stratification of the envenomation. It is administered in moderate and high-risk cases, while symptomatic medication and clinical observation are advised in low-risk cases^11^. This advice was provided to the healthcare team by the Toxicological Information Centre, but the victim had already received the antitoxin dose in the emergency department. This behavior shows the need for increased training of health professionals, so that envenomation**s** caused by venomous animals can be handled according to standardized protocols. It also highlights the importance of toxicological information centers in guiding treatment of such cases. 

## CONCLUSION

This is the first described case of scorpionism caused by *T. serrulatus* as described by Lutz & Mello, 1922 (Scorpiones, Buthidae). It is a species non-native to the state of Pará (North region), Amazonian region. The species originated in the state of Bahia (Northeast) and was transported by truck (amongst fruit) and was responsible for a low-risk scorpionism incident. If the species were to be introduced in the northern region, it could cause ecological disruption and may pose a threat to the local homeostasis and public health. Hence, close surveillance of invading species of the Amazonian Scorpio fauna is important, as is the need for public health projects to improve the management of envenomation and prevent future accidents.
